# *Mycobacterium chimaera* in Heater–Cooler Units in Denmark Related to Isolates from the United States and United Kingdom 

**DOI:** 10.3201/eid2303.161941

**Published:** 2017-03

**Authors:** Erik Svensson, Elsebeth Tvenstrup Jensen, Erik Michael Rasmussen, Dorte Bek Folkvardsen, Anders Norman, Troels Lillebaek

**Affiliations:** Statens Serum Institut, Copenhagen, Denmark

**Keywords:** mycobacterium, nontuberculous mycobacteria, *Mycobacterium avium* complex, single-nucleotide polymorphism, water, bacterial genetics, environmental microbiology, molecular epidemiology, molecular typing, tuberculosis and other mycobacteria, bacteria

## Abstract

*Mycobacterium chimaera* was present at high rates (>80%) in heater–cooler units (HCUs) from all 5 thoracic surgery departments in Denmark. Isolates were clonal to HCU-associated isolates from the United States (including some from patients) and United Kingdom. However, *M. chimaera* from 2 brands of HCU were genetically distinct.

Based on reports from 2015 (*1*,*2*), the European Centre for Disease Prevention and Control issued a Rapid Risk Assessment alert on April 30, 2015, associating invasive cardiovascular infections with *Mycobacterium chimaera* in water tanks of heater–cooler units (HCUs) used during open-chest heart and vascular surgery (*3*). Subsequently, additional cases from Europe (*4*) and the United States potentially associated with HCUs have been described (*5*–*7*). Preliminary data indicate that the isolates from the patients, the HCUs in hospitals, and the HCUs at the manufacturer are similar (*8*). The aim of this study was to determine *M. chimaera* prevalence in Denmark HCUs and, if present, phylogenetically characterize and quantify the strains.

## The Study

Statens Serum Institut, the Danish Patient Safety Authority, and the Danish Medicines Agency decided to investigate all the HCUs in Denmark. Approval from a human or animal research ethics board was not required to conduct this study. The infection prevention control units and thoracic surgery department staff from 5 local hospitals were instructed to collect water and biofilm samples from the HCUs and send them to Statens Serum Institut for testing. In brief, a water culture method adapted for low concentrations of mycobacteria with high concentrations of contaminants was used, and mycobacterial isolates were identified by internal transcribed spacer sequencing (Technical Appendix). One *M. chimaera* isolate from each thoracic surgery department and one unrelated patient isolate were subjected to whole-genome sequencing (WGS). A total of 10 million paired-end Illumina sequencing reads (Illumina Denmark ApS, Copenhagen, Denmark) were deposited in the European Nucleotide Archive under study number PRJEB18427.

*M. chimaera* was found in 18/21 (86%) HCUs, representing all 5 thoracic surgery departments in Denmark (Table). Four sites used the Sorin 3T HCU (Sorin Group, Arvada, CO, USA); 14/16 (88%) units contained *M. chimaera*. One site used Maquet brand HCUs (Maquet, Wayne, NJ, USA), 4/5 (80%) units contained *M. chimaera*. The strain *M. gordonae* was found irregularly throughout the HCUs (Table). Both water and biofilm samples could be cultured and were equally effective for the detection of mycobacteria. We used the filter culture method for quantitative analysis purposes and for a simpler workflow. However, the quantitative culture results were poor quality because the analytic sensitivity was low and many samples were heavily contaminated (Table; Technical Appendix Figure).

**Table:** Identification of *Mycobacterium* spp. from water and biofilm samples taken from heater–cooler units from 5 heart surgery centers, Denmark, July–October 2015*

Heater–cooler unit	Water sample results	Biofilm sample results	Quantitative culture, CFU/L†
A1	*M. chimaera*	NA	100
A2	*M. chimaera*	NA	Mold
A3	*M. chimaera*	NA	60
B1	*M. chimaera*	NA	Mold
C1	*M. chimaera*	*M. chimaera*	0
C2	*M. gordonae*	*M. gordonae*	9
C3	*M. chimaera*	*M. chimaera*	Mold
C4	*M. gordonae*	*M. gordonae*	57
D1	*M. chimaera*	*M. chimaera*	>1,000
D2	*M. chimaera*	NA	Mold
D3	*M. chimaera*	*M. chimaera*	Mold
D4	*M. chimaera*	*M. chimaera*	>1,000
D5	*M. chimaera*	*M. chimaera*	Mold
D6	*M. chimaera*	NA	Mold
D7	*M. chimaera*	*M. chimaera*	Mold
D8	*M. chimaera*	*M. chimaera*	Mold
E1	*M. gordonae*	*M. chimaera*	0
E2	*M. chimaera*	*M. chimaera*, *M. gordonae*	0
E3	*M. chimaera*	*M. chimaera*	0
E4	*M. chimaera*	*M. chimaera*, *M. gordonae*	300
E5	No growth	NA	0

*NA, not available. †Bacterial concentration of the original sample.

WGS analysis (Figure) showed that the 4 isolates from the Denmark Sorin 3T HCUs were nearly identical (<3 single nucleotide polymorphisms [SNPs]). Conversely, the isolate collected from the Maquet HCU was genetically distinct, showing 47–49 SNP differences compared with the isolates from the Sorin 3T HCUs. The unrelated patient isolate was not closely related to the HCU isolates (30–37 SNP differences). 

**Figure:**
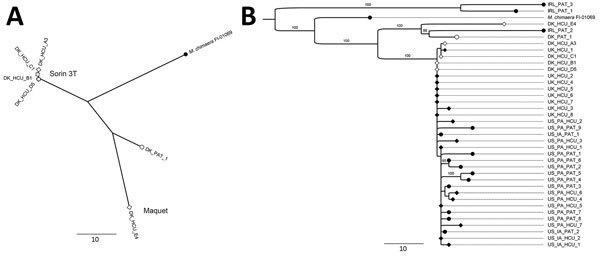
Maximum parsimony phylogenies showing the relationship between *Mycobacterium chimaera* isolates found in patients (circles) and heater–cooler units (HCUs; diamonds) in Denmark. *M. chimaera* strain FI-0169 (accession no. PRJNA356276) was included for reference. A) Tree showing isolates from Sorin 3T (Sorin Group, Arvada, CO, USA) and Maquet (Maquet, Wayne, NJ, USA) HCUs and a non–HCU-associated isolate from a patient (PAT) in Denmark (DK). B) Phylogenetic tree comparing isolates from Denmark to 31 isolates collected in 3 other countries (Ireland [IRL], United Kingdom, and United States) and retrieved from the European Nucleotide Archive (http://www.ebi.ac.uk/ena). Isolates from Denmark are indicated by open symbols and isolates from other countries by solid symbols. Branch values indicate percentwise bootstrap support (only >70% support is shown), based on 100 replicates. IA, Iowa; PA, Pennsylvania. Scale bars represent a difference of 10 single-nucleotide polymorphisms.

We compared the sequences we collected with 31 other sequenced *M. chimeaera* isolates previously collected (2009–2016) and available in the European Nucleotide Archive (http://www.ebi.ac.uk/ena). This dataset comprised 3 non–HCU-associated patient isolates from Ireland; 9 Sorin 3T HCU isolates and 11 HCU-associated patient isolates from Pennsylvania and Iowa, USA (*6*); and 8 HCU water sample isolates from the United Kingdom (accession nos. PRJNA294775, PRJNA344472, PRJNA345021, and PRJNA324238 for the 4 groups, respectively). Unexpectedly, the *M. chimaera* sequences from the Denmark Sorin 3T HCUs were nearly identical to the isolates from the United States and United Kingdom (median difference 3 SNPs; interquartile range [IQR] 1–5 SNPs) and were similar to all Sorin 3T-associated patient isolates (median difference 6 SNPs; IQR 3–9 SNPs). We saw a distinctly closer relationship between the isolates from Denmark Sorin 3T HCUs and the isolates from UK and US HCUs than between Denmark Sorin 3T HCUs and the unrelated Denmark or Ireland patient isolates or the Denmark Maquet HCU isolate (Figure). 

Overall, the 32 isolates associated with the Sorin 3T HCUs (online Technical Appendix Table) were found to have 15 common SNPs and 0–18 SNP differences between any 2 isolates (median difference 5 SNPs; IQR 3–8 SNPs). These findings support the conclusion by Haller et al. that *M. chimaera* from the Sorin 3T HCUs have a common source (*8*). The *M. chimaera* sequences from the UK HCU water samples were genetically nearly identical to the US and Denmark isolates; we therefore conclude that the UK isolates also originated from Sorin 3T HCUs.

No patients with *M. chimaera* infections associated with open-chest surgery have been suspected or detected in Denmark. Searching the International Reference Laboratory of Mycobacteriology database, which includes all mycobacteria cultures in Denmark, from 1991 to 2016, we found no records of *M. avium* complex isolates from patients that had an open-chest operation. 

Following our findings, 1 thoracic surgery department decided to keep the HCUs in the operating theater but encased them in housings with separate ventilation. Two departments were unable to take the HCUs out of the theaters but decided to move the HCUs as distant as possible from the patients and decontaminate more frequently. Two of the departments had their HCUs outside the operating room already and therefore kept their policies regarding HCUs.

## Conclusions

We found that *M. chimaera* was present in most HCUs in Denmark. Isolates from Sorin 3T brand HCUs were identical to the HCU isolates from the United States and the United Kingdom, and thus they appear to have the same origin. Because all 5 of the thoracic surgery departments in Denmark had contaminated HCUs and because mycobacterial contamination has been reported in multiple published studies during 2015–2016 (*4*–*6*), we find it likely that most Sorin 3T HCUs made in the past 8–10 years potentially are contaminated by the same *M. chimaera* strain. In addition, because 80% of the Maquet HCUs also contained *M. chimaera*, although phylogenetically different from the Sorin 3T strains, we suggest mycobacterial contamination might be a general problem for HCUs.

Technical Appendix Materials and methods, whole-genome sequencing information on *Mycobacterium chimaera* isolates, and quantitative culture plates showing growth of isolates on filters.
