# Supplementary material for: Mycobacterium chimaera in Heater–Cooler Units in Denmark Related to Isolates from the United States and United Kingdom
Source: Emerg Infect Dis. 2017 Mar;23(3):507–9. doi: 10.3201/eid2303.161941 (PMC5382728; doi:10.3201/eid2303.161941)
Supplement: Technical Appendix — Materials and methods, whole-genome sequencing information on Mycobacterium chimaera isolates, and quantitative culture plates showing growth of isolates on filters. [file 16-1941-Techapp-s1.pdf]

Article DOI: http://dx.doi.org/10.3201/eid2303.161941

Mycobacterium chimaera in Heater–Cooler Units in Denmark Related to Isolates from the United States and United Kingdom

Technical Appendix

Sampling

Statens Serum Institut, the Danish Patient Safety Authority, and the Danish Medicines Agency decided to investigate all Denmark heater–cooler units (HCUs) and to increase the awareness of symptoms of *M. chimaera* infections in patients who had undergone open chest heart surgery previously. The 5 local hospital's infection prevention and control units, surgeons, perfusionists, and other relevant staff were specifically recruited, participated in network meetings, and were provided with instructions on how to sample water and biofilm from the HCUs. In return, Statens Serum Institut and the Danish Medicines Agency gained helpful information on the number and models of HCUs used throughout the country.

Samples were collected from all the HCUs located in thoracic surgery departments (n = 5) throughout the country. The departments were instructed to submit 1 L of water from each tank for the detection of planktonic mycobacteria, as well as provide swabs from the inner surfaces of tube mouths from each HCU for the detection of mycobacteria in the biofilm.

Culture

Each 1-L water sample was divided into 3 aliquots. All 3 were concentrated by centrifugation at 3,000 × *g* in a Sorvall RC 6 Plus Centrifuge (Thermo Fisher Scientific, Waltham, MA, USA), followed by removal of the supernatant. The sediments were decontaminated separately using N-acetyl-cysteine and 2% NaOH (Mycoprep, Becton Dickinson, Sparks, MD, USA) for 10 min, neutralized with phosphate buffer (pH 6.8), and centrifuged for 15 min at 3,700 rpm in a Heraeus Multifuge 3SR+ (Thermo Fisher Scientific Waltham, MA, USA). Then supernatants were removed, leaving ≈1.5 mL of sediment. Two of

the 3 sediment aliquots were used for qualitative culture and the remaining aliquot for quantitative culture on a filter. A portion from each of the 2 aliquots was inoculated separately into a mycobacterium growth indicator tube (Becton Dickinson, Sparks, MD, USA) and a Löwenstein-Jensen tube (SSI Diagnostica, Copenhagen, Denmark). The remaining portion of the 2 aliquots were kept in the refrigerator as a backup if the culture was contaminated. The third aliquot was frozen for later quantitative culture. To quantitate, the frozen aliquots were thawed and diluted with 100 mL of phosphate buffer and passed through a MicroFunnel 300 black membrane filter with a 0.45-µm pore size (Pall Corporation, Westborough, MA, USA). The filter was put on Middlebrook 7H11 agar and incubated at 30°C for 8 weeks.

Identification

The growing mycobacteria were identified using GenoType CM line probe assay (Hain Lifescience, Nehren, Germany) as a screening test. If the test result was positive for *M. intracellulare*, we then performed internal transcribed spacer (ITS) sequencing (*1*,*2*).

Five microliters of DNA (extracted from cells with heat inactivation and sonication) was amplified and hybridized with GenoType CM kit (Hain Lifescience) according to the instructions from the manufacturer. Initial PCR for ITS sequencing was done with a 5 µL sample of DNA in a 50 µL reaction containing 5 µL 10× PCR buffer, 10 µL 5× Q-solution, 2 µL 25 mM $MgCl_2$, 1 µL 10 µM primer ITS_F, 1 µL 10 µM primer ITS_R (*1*,*2*), 0.25 µL HotStarTaq (HotStarTaq Master Mix Kit; QIAGEN, Hilden, Germany), and water (DNase, RNase, and protease free). The thermal profile involved a denaturation step of 95°C for 15 min; 40 cycles of 95°C for 60 s, 59°C for 60 s, and 72°C for 60 s; a final extension at 72°C for 10 min; and a cool down at 4°C. The sequencing was performed using the Big Dye Terminator v1.1 Cycle Sequencing Kit (Applied Biosystems, ThermoFisher Scientific, Waltham, MA, USA) according to the manufacturer's protocol. Sequence analysis was performed using Sequencing Analysis Software v.5.3.1 (Applied Biosystems).

Whole-Genome Sequencing

In brief, 1 mL of a culture enriched in Dubos broth (SSI Diagnostika, Hilleroed, Denmark) was centrifuged at 13,000 rpm for 10 min, the supernatant removed, and the pellet

resuspended in 300 µL TE buffer (0.01 M Tris-HCl, 0.001 M EDTA, pH 8.0). The resuspended pellets were heated at 80°C for 20 min to kill the cells and incubated with 50 µL of 10 mg/mL lysozyme for 24 h at 37°C on a Thermomixer C (Eppendorf, Hamburg, Germany). Then 70 µL of 10% sodium dodecyl sulfate and 5 µL of 10 mg/mL proteinase K were added, and the mixture was incubated for 10 min at 65°C. Next, 100 µL of 5 M NaCl and 100 µL of N-cetyl-N,N,N-trimethyl ammonium bromide/NaCl was added. The tubes were vortexed briefly and incubated for 10 min at 65°C. An equal volume of chloroform/isoamyl alcohol (24:1, vol/vol) was added, the mixture was vortexed for 10 s, and centrifuged at 13,000 rpm for 10 min. Afterwards, 0.6 volume of isopropanol was added to the supernatant to precipitate the DNA. The tubes were left overnight at –20°C and centrifuged for 15 min; the pellet was washed once with 70% ethanol, air-dried, and redissolved in 20 µL of TE buffer.

DNA was normalized to a concentration of 0.5 ng/µL using a Biomek NX Lab Automation Workstation (Beckman Coulter, Indianapolis, IN, USA). DNA libraries were generated using a Nextera XT DNA preparation kit (Illumina, Inc., San Diego, CA, USA) according to the manufacturer’s instructions but diluted an extra 4-fold before being loaded on to the NextSeq 500 platform (Illumina Inc.) with 2×150 bp paired-end chemistry.

Paired-end Illumina reads were end-trimmed (base-quality >Q3; minimum end-trimmed length >35 bp) and filtered using Trimmomatic (http://www.usadellab.org/cms/?page=trimmomatic), and mapped to the 5.8 Mbp *M. chimera* FI-01069 chromosome sequence (accession no. PRJNA324238) using the Burrows-Wheeler Alignment tool bwa (http://bio-bwa.sourceforge.net/) (see Technical Appendix Table for an overview of mapped samples used in this study). Variant calling was performed with SAMtools and BCFtools (http://www.htslib.org/) (*3*). Single-nucleotide polymorphisms (SNPs) were filtered according to the following criteria: having a phred-scaled variant calling quality exceeding 20 (http://gatkforums.broadinstitute.org/gatk/discussion/4260/phred-scaled-quality-scores) and being supported by ≥5 reads with a frequency of 85% in ≥1 sample. However, to minimize the influence of horizontal gene transfer, regions in which ≥3 SNPs occurred together within 100 bp were excluded from the analysis. For individual samples, SNPs were considered as fixated if they were supported by ≥3 reads with a SNP-frequency above 70%, or otherwise called the reference, unless present in ≥5 other samples. Phylogenetic comparison of concatenated SNPs was performed with the Seaview program (*4*) by using the built-in maximum parsimony

function (Dnapars, http://evolution.genetics.washington.edu/phylip/doc/dnapars.html). Branch support values were calculated using 100 bootstrap replicates. A distance matrix was created by importing the generated SNP alignment in FASTA format (http://zhanglab.ccmb.med.umich.edu/FASTA/) into the statistics software RStudio (v1.0.44) (https://www.rstudio.com/) using the Analyses of Phylogenetics and Evolution package (http://ape-package.ird.fr/) to calculate pairwise sample distances.

Technical Appendix Table. Overview of whole-genome sequencing of *Mycobacterium chimaera* isolated from heater–cooler units (HCUs) and patients, Denmark, July–October 2015*

Sample name	Study accession no.	Run accession no.	Country	Collection date	Isolation source	No. trimmed reads	Reads mapped, %	FI-01069 coverage, X
DK_HCU_A3	PRJEB18427	ERR1744899	DK	2015 Jul 15	HCU (3T)	422,370	88.3	9
DK_HCU_B1	PRJEB18427	ERR1744900	DK	2015 Aug 8	HCU (3T)	2,916,088	88.7	61
DK_HCU_C1	PRJEB18427	ERR1744901; ERR1744902	DK	2015 Jul 28	HCU (3T)	2,011,606	87.7	41
DK_HCU_D5	PRJEB18427	ERR1744903	DK	2015 Oct 1	HCU (3T)	1,552,694	85.9	32
DK_HCU_E4	PRJEB18427	ERR1744904	DK	2015 Oct 1	HCU (Maquet)	1,850,980	87.4	39
DK_PAT_1	PRJEB18427	ERR1744905	DK	2015 Jul 13	BAL	1,034,652	90.0	23
FI-01069	PRJEB18427	ERR1744906	IT	2001	BAL	1,636,840	96.1	37
IRL_PAT_1	PRJNA294775	SRR2338871	IRL	2009	Sputum	3,466,168	92.5	52
IRL_PAT_2	PRJNA294775	SRR2338873	IRL	2013	Sputum	4,040,276	96.4	81
IRL_PAT_3	PRJNA294775	SRR2338874	IRL	2014	BAL	3,334,536	90.3	70
UK_HCU_WAT_1	PRJNA324238	SRR4068047	UK	2015	HCU (Unknown)	2,122,042	90.3	46
UK_HCU_WAT_2	PRJNA324238	SRR4119603	UK	2015	HCU (Unknown)	2,642,246	92.5	39
UK_HCU_WAT_3	PRJNA324238	SRR4119606	UK	2015	HCU (Unknown)	3,459,964	87.2	49
UK_HCU_WAT_4	PRJNA324238	SRR4119612	UK	2015	HCU (Unknown)	2,753,690	92.6	41
UK_HCU_WAT_5	PRJNA324238	SRR4119617	UK	2015	HCU (Unknown)	2,762,350	92.3	41
UK_HCU_WAT_6	PRJNA324238	SRR4119619	UK	2015	HCU (Unknown)	2,604,538	93.0	39
UK_HCU_WAT_7	PRJNA324238	SRR4119623	UK	2015	HCU (Unknown)	7,623,682	88.2	104
UK_HCU_WAT_8	PRJNA324238	SRR4119656	UK	2015	HCU (Unknown)	5,384,290	91.2	79
US_PA_HCU_1	PRJNA344472	SRR4295156	USA (PA)	2015 Jul 25	HCU (3T)	2,514,644	94.5	90
US_PA_HCU_2	PRJNA344472	SRR4295157	USA (PA)	2015	HCU (3T)	1,996,066	88.0	65
US_PA_PAT_1	PRJNA344472	SRR4295158	USA (PA)	2015 May 1	Human	2,472,668	94.5	90
US_PA_HCU_3	PRJNA344472	SRR4295159	USA (PA)	2015 Oct 30	HCU (3T)	2,861,298	90.3	97
US_PA_PAT_2	PRJNA344472	SRR4295160	USA (PA)	2015 Sep 11	Human	2,934,922	94.7	106
US_PA_PAT_3	PRJNA344472	SRR4295161	USA (PA)	2015 Nov 6	Human	2,184,438	91.1	77
US_PA_PAT_4	PRJNA344472	SRR4295162	USA (PA)	2016 Feb 3	Human	2,636,322	91.4	94
US_PA_PAT_5	PRJNA344472	SRR4295163	USA (PA)	2016 Mar 21	Human	2,112,644	90.9	76
US_PA_HCU_4	PRJNA344472	SRR4295164	USA (PA)	2015	HCU (3T)	3,176,156	90.0	108
US_PA_HCU_5	PRJNA344472	SRR4295165	USA (PA)	2015	HCU (3T)	3,143,036	93.6	110
US_PA_HCU_6	PRJNA344472	SRR4295166	USA (PA)	2015	HCU (3T)	2,731,838	94.6	99
US_PA_PAT_6	PRJNA344472	SRR4295167	USA (PA)	2015 Jun 15	Human	3,030,308	93.9	109
US_PA_PAT_7	PRJNA344472	SRR4295168	USA (PA)	2014 Jul 24	Human	2,595,368	94.7	94
US_PA_PAT_8	PRJNA344472	SRR4295169	USA (PA)	2014 May 20	Human	2,309,386	94.4	84
US_PA_HCU_7	PRJNA344472	SRR4295170	USA (PA)	2015 Aug 19	HCU (3T)	2,139,370	86.8	72
US_PA_PAT_9	PRJNA344472	SRR4295171	USA (PA)	2015 Jan 28	Human	2,115,298	87.9	72
US_IA_PAT_1	PRJNA345021	SRR4324922	USA (IA)	2015 Nov 11	Blood	1,230,194	95.4	44
US_UA_PAT_2	PRJNA345021	SRR4324923	USA (IA)	2016 Feb 8	Blood	2,087,698	92.7	89
US_IA_HCU_1	PRJNA345021	SRR4324924	USA (IA)	2016 Jan 28	HCU (3T)	1,173,912	87.0	43
US_IA_HCU_2	PRJNA345021	SRR4324925	USA (IA)	2016 Apr 13	HCU (3T)	1,524,522	93.5	72

*DK, Denmark; HCU, heater–cooler unit; IA, Iowa; IRL, Ireland; IT, Italy; PA, Pennsylvania; PAT, patient; WAT, water.

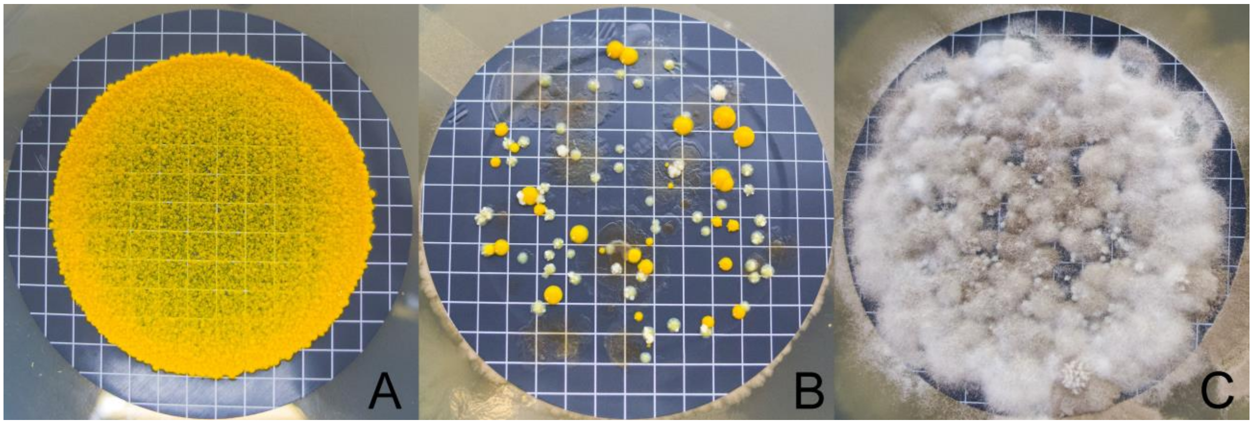

Technical Appendix Figure. Quantitative culture of filtered water from heater–cooler units. The images show filters with (A) >1,000 CFU/L, (B) 300 CFU/L with some contamination from other bacteria, and (C) heavy overgrowth of mold.

References

1. De Smet KA, Brown IN, Yates M, Ivanyi J. Ribosomal internal transcribed spacer sequences are identical among *Mycobacterium avium*-intracellulare complex isolates from AIDS patients, but vary among isolates from elderly pulmonary disease patients. Microbiology. 1995;141:2739–47. PubMed http://dx.doi.org/10.1099/13500872-141-10-2739

2. Frothingham R, Wilson KH. Sequence-based differentiation of strains in the *Mycobacterium avium* complex. J Bacteriol. 1993;175:2818–25. PubMed http://dx.doi.org/10.1128/jb.175.10.2818-2825.1993

3. Li H. A statistical framework for SNP calling, mutation discovery, association mapping and population genetical parameter estimation from sequencing data. Bioinformatics. 2011;27:2987–93. PubMed http://dx.doi.org/10.1093/bioinformatics/btr509

4. Gouy M, Guindon S, Gascuel O. SeaView version 4: a multiplatform graphical user interface for sequence alignment and phylogenetic tree building. Mol Biol Evol. 2010;27:221–4. PubMed http://dx.doi.org/10.1093/molbev/msp259
